# Adherence in a randomised controlled trial comparing liberal and restrictive red blood cell (RBC) transfusion protocols after cardiac surgery (TITRe2)

**DOI:** 10.1186/1745-6215-12-S1-A131

**Published:** 2011-12-13

**Authors:** Katie Pike, Rachel Brierley, Chris A Rogers, Gavin J Murphy, Barney C Reeves

**Affiliations:** 1 Bristol Heart Institute, University of Bristol, Bristol, BS2 8HW, UK

## Objectives

The TITRe2 trial is comparing two haemoglobin (Hb) thresholds for RBC transfusion after cardiac surgery, Hb<9.0g/dl (liberal) vs. Hb<7.5g/dl (restrictive). Based on historic data, and with complete adherence, transfusion rates should be 100% in the liberal and 30% in the restrictive group. Convergence of these rates due to non-adherence severely threatens the power of the trial; there is also concern about differential non-adherence, with transfusion being delayed or withheld in the liberal group when Hb remains close to the 9.0g/dl threshold.

## Methods

In order to capture non-adherence, research staff collect data describing:

• The lowest daily Hb;

• Date and time of each RBC transfusion and the preceding Hb measurement;

• Number of breaches of the allocated threshold before a transfusion is prescribed.

These data allow non-adherence to the randomisation and transfusion protocols to be detected:

• Failure to randomise when the 9.0g/dl threshold is breached;

• Randomised >24 hours after first breaching Hb 9.0g/dl threshold;

• Randomised without or before breaching Hb 9.0g/dl threshold;

• After randomisation, transfusion given when allocated threshold not breached (‘extra’), or transfusion withheld when allocated threshold breached (‘withheld’);

Instances of extra and withheld transfusions are classified as mild, moderate or severe depending on their likely influence on overall transfusion rates.

## Results

56% of participants are being randomised; about 8% of the remaining 44% consented participants breach the 9.0g/dl threshold but are not randomised. 3% of randomised participants are randomised >24 hours after first breaching, but none has been randomised without or before breaching the 9.0g/dl threshold. 32% of participants have had ≥1 instance of non-adherence to the transfusion protocol; in 6%, non-adherence was judged severe (extra – transfused and patient did not breach at any point post-randomisation; withheld – not transfused and patient had no post-randomisation transfusions). Site-specific rates of non-adherence are being fedback to try to improve adherence. Rates of transfusion in the liberal and restrictive groups are confidential to the Data Monitoring and Ethics Committee (DMEC).

## Conclusions

We believe that this is the first attempt to measure withheld transfusions in trials of this kind. Data collection to do this is burdensome but satisfactory. The current rates of transfusion in the liberal and restrictive groups are, so far, judged by the DMEC to be consistent with the sample size justification.

